# Cholecystectomy is associated with higher risk of early recurrence and poorer survival after curative resection for early stage hepatocellular carcinoma

**DOI:** 10.1038/srep28229

**Published:** 2016-06-20

**Authors:** Tao Li, Shu-Kang Wang, Xu-Ting Zhi, Jian Zhou, Zhao-Ru Dong, Zong-Li Zhang, Hui-Chuan Sun, Qing-Hai Ye, Jia Fan

**Affiliations:** 1 Department of General Surgery, Qilu Hospital, Shandong University, Jinan, P.R. China; 2 Department of Biostatistics, School of Public Health, Shandong University, Jinan, P.R. China; 3 Liver Cancer Institute, Zhongshan Hospital, Fudan University, Shanghai, P.R. China

## Abstract

Although cholecystectomy has been reported to be associated with increased risk of developing hepatocellular carcinoma (HCC), the association between cholecystectomy and prognosis of HCC patients underwent curative resection has never been examined. Through retrospective analysis of the data of 3933 patients underwent curative resection for HCC, we found that cholecystectomy was an independent prognostic factor for recurrence-free survival (RFS) of patients at early stage (BCLC stage 0/A) (p = 0.020, HR: 1.29, 95% CI: 1.04–1.59), and the 1-, 3-, 5-year RFS rates for patients at early stage were significantly worse in cholecystectomy group than in non-cholecystectomy group (80.5%, 61.8%, 52.0% vs 88.2%, 68.8%, 56.8%, p = 0.033). The early recurrence rate of cholecystectomy group was significantly higher than that of non-cholecystectomy group for patients at early stage (59/47 vs 236/333, p = 0.007), but not for patients at advanced stage (BCLC stage C) (p = 0.194). Multivariate analyses showed that cholecystectomy was an independent risk factor for early recurrence (p = 0.005, HR: 1.52, 95% CI: 1.13–2.03) of early stage HCC, but not for late recurrence (p = 0.959). In conclusion, cholecystectomy is an independent predictor for early recurrence and is associated with poorer RFS of early stage HCC. Removal of normal gallbladder during HCC resection may be avoided for early stage patients.

For more than a century cholecystectomy has been a method of choice in surgical management of gallbladder diseases, and the declining incidence of gallbladder cancer is considered to related to the increasing cholecystectomy rates^1^. Recently, much more epidemiological investigations and meta-analyses have addressed the relation between cholecystectomy and hepatocellular carcinoma (HCC)^2^,^3^,^4^,^5^. Though a causal association between them remains to be established, the majority of these studies indicate that cholecystectomy is associated with an increased risk of developing HCC.

The reason for increased risk of developing HCC after cholecystectomy is sill unclear. One potential mechanism may involve chronic inflammation. Cholecystectomy can not only cause dilation of the CBD, elevated bile duct pressure, subsequent cholestasis and infection^6^,^7^,^8^, but also can induce systemic inflammation and cause increase of postoperative Interleukin (IL)-1, IL-6 and C-reactive protein (CRP)^9^,^10^,^11^. The link between chronic inflammation and HCC is well established^12^. Persistent inflammation will not only cause necrosis and regeneration of hepatocytes, thereby lead to DNA instability in the hepatocytes, and cause HCC to occur more frequently^13^,^14^,^15^, but also stimulate the release of cytokines, chemokines, vascular adhesion molecules and reactive oxygen species^5^,^16^,^17^,^18^, all of which play decisive roles in cancer development.

Previous studies of HCC have revealed various risk factors related to postoperative outcome^19^,^20^,^21^. The severity of virally induced inflammation, which was well correlated with viral serostatus, has been established as an adverse risk factor for postoperative recurrence^22^,^23^,^24^, and antiviral therapy during the surveillance period is associated with reduced recurrence and improvement in overall survival in HBV-related HCC^25^,^26^. The decreased incidence of recurrence in patients who have used nonsteroidal anti-inflammatory drugs (NSAIDs), regardless of patients’ viral hepatitis status, is supportive of a role for non-virus related inflammation in HCC recurrence^27^. Since an association is biologically plausible, the relation between cholecystectomy and outcome of HCC merits investigation. In this study, we retrospectively analyzed the data of 3933 consecutive HCC patients treated by curative resection, to investigate the impact of cholecystectomy on postoperative recurrence and survival of HCC patients.

## Results

### Patient distribution, survival and recurrence rates according to BCLC staging

A total of 3933 HCC patients received curative resection for HCC (The clinicopathologic characteristics of all 3933 HCC patients are listed in supplementary Table 1), among them, 639 patients also underwent cholecystectomy simultaneously. Of these 639 patients, 71 patients had gallbladder disease: acute cholecystitis in 1 patient; adenomyoma in 13 patients; gallstones in 40 patients; polyp in 8 patients; and HCC invasion or metastasis of the gallbladder in 9 patients. The overall prevalence of gallbladder invasion or metastasis from HCC was 0.2% (9/3933). Of the other 568 patients with normal gallbladder removed, 99 patients underwent right hemihepatectomy, 85 patients underwent left hemihepatectomy, 28 patients underwent central hepatectomy, 356 patients underwent local or segment resection and the gallbladder was removed because the tumor was adjacent to the gallbladder. All patients were followed up and the median follow-up time was 20 months (range, 1–168 months).

Further tumor staging according to the Barcelona Clinic Liver Cancer (BCLC) staging system revealed that the ratio of patients at early stage (BCLC stage 0/A) was significantly higher in no-cholecystectomy group than in cholecystectomy group (61.2% vs 48.0%, p < 0.001, Table 1). Though the ratio of patients received major resection was significantly higher in cholecystectomy group than in no-cholecystectomy group (283/639 vs 1006/3294, p < 0.001, Table 1), there were no significant differences regarding postoperative complications including bile leakage (p = 0.478), hemorrhage (p = 0.739), intra-abdominal abscess (p = 0.373) and pleural effusion (p = 0.233).

The incidence of intrahepatic recurrence of cholecystectomy group was significantly higher than that of no-cholecystectomy group (1072/3294 vs 251/639, p = 0.001). The early recurrence rate of cholecystectomy group were also significantly higher than that of no-cholecystectomy group (65.7% vs 53.5%, p < 0.001, Table 1). However, when patients were stratified according to BCLC stage, the difference was significant only for patients at early stage (p = 0.007, Table 1) or intermediate stage (BCLC stage B) (p = 0.048, Table 1), but not for patients at advanced stage (BCLC stage C) (p = 0.194, Table 1).

**Figure 1:**
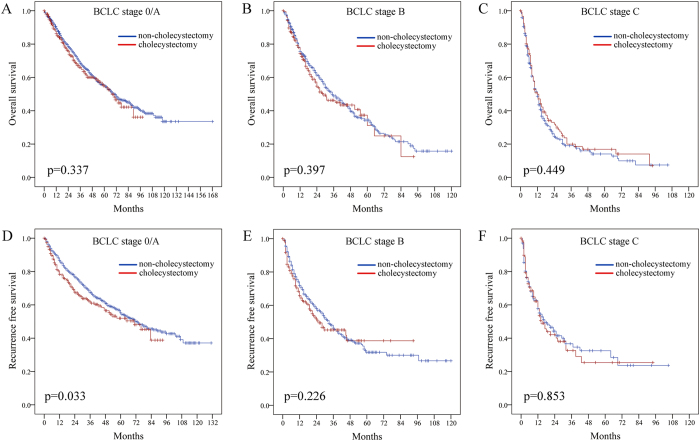
(**A–C**) Comparison of cumulative incidence of OS according to BCLC stage between cholecystectomy group and non-cholecystectomy group; (**D–F**) Comparison of cumulative incidence of RFS according to BCLC stage between cholecystectomy group and non-cholecystectomy group.

Stratified analysis of overall survival (OS) according to BCLC staging system demonstrated no significant difference between the cholecystectomy group and no-cholecystectomy group at early, intermediate or advanced stage (Fig. 1A–C, p = 0.337, 0.397 and 0.449, respectively), and only for patients at early stage, the recurrence free survival (RFS) rates of cholecystectomy group were significantly worse than those of non-cholecystectomy group (80.5%, 61.8%, 52.0% vs 88.2%, 68.8%, 56.8%, Fig. 1D, p = 0.033). The RFS of patients at intermediate or advanced stage did not differ significantly between the cholecystectomy group and no-cholecystectomy group (Fig. 1E,F, p = 0.226, 0.853, respectively).

### Comparison of clinicopathologic characteristics between early stage HCC patients in no-cholecystectomy group and cholecystectomy group

The clinicopathologic characteristics of early stage HCC patients in no-cholecystectomy group and cholecystectomy group were compared in Table 2. Despite age and tumor size, there were no significant differences between these two groups regarding serum level of AFP (792/1223 vs 113/194, p = 0.403), ALT (1771/244 vs 260/47, p = 0.115) and GGT (910/1105 vs 124/183, p = 0.117). There were also no significant differences regarding the incidence of hepatitis B virus infection (298/1717 vs 47/260, p = 0.811) and cirrhosis (299/1716 vs 49/258, p = 0.608) between these two groups for early stage patients.

### Univariate and multivariate analysis of risk factors related to RFS of early stage HCC patients

Since for patients at early stage, the RFS rates of cholecystectomy group were significantly worse than those of non-cholecystectomy group, we further investigated whether cholecystectomy was an independent prognostic factor for RFS of patients at early stage. Table 3 demonstrated the prognostic factors related to postoperative RFS of early stage HCC patients in univariate and multivariate analysis. Multivariate analysis revealed that despite HBsAg (p = 0.031, HR: 1.30, 95% CI: 1.02–1.65), AFP level (p < 0.001, HR: 1.39, 95% CI: 1.18–1.64), GGT level (p = 0.005, HR: 1.25, 95% CI: 1.07–1.47), tumor size (p = 0.002, HR: 1.29, 95% CI: 1.10–1.52), postoperative TACE (p < 0.001, HR: 3.39, 95% CI: 2.89–3.99), cholecystectomy (p = 0.020, HR: 1.29, 95% CI: 1.04–1.59) was also an independent prognostic factor for RFS of early stage HCC patients.

### Univariate and multivariate analysis of predictive factors for early intrahepatic recurrence of early stage HCC patients

Univariate analysis of predictive factors for early and late intrahepatic recurrence of early stage HCC patients were listed in Table 4 and Table 5 respectively. Univariate analysis revealed that cholecystectomy was a risk factor for early recurrence (p < 0.001, HR: 1.69, 95% CI: 1.27–2.24), but had no predictive significance for late recurrence (p = 0.893, HR: 1.03, 95% CI: 0.68–1.55). Multivariate analysis (Table 4) revealed that besides AFP level (p = 0.001, HR: 1.54, 95% CI: 1.18–2.01), GGT level (p = 0.002, HR: 1.49, 95% CI: 1.16–1.92), tumor capsule (p = 0.016, HR: 1.33, 95% CI: 1.05–1.69) and tumor size (p < 0.001, HR: 1.78, 95% CI: 1.40–2.27), postoperative TACE (p < 0.001, HR: 3.67, 95% CI: 2.85–4.71) and IFN-α treatment (p = 0.010, HR: 1.89, 95% CI: 1.17–3.05), cholecystectomy (p = 0.005, HR: 1.52, 95% CI: 1.13–2.03) was also an independent risk factor for early recurrence of early stage HCC patients. In addition, for early stage patients that received minor resection, cholecystectomy still was an independent risk for early recurrence (p = 0.048, HR: 1.47, 95% CI: 1.00–2.15, Supplementary Table 2). Only liver cirrhosis (p = 0.001, HR: 2.24, 95% CI: 1.40–3.57, Table 5) and postoperative TACE (p < 0.001, HR: 2.94, 95% CI: 2.20–3.83, Table 5) were independent risk factors for late recurrence of early stage HCC patients.

## Discussion

HCC is a clear example of inflammation-related cancer as more than 90% of HCC arises in the context of hepatic injury and inflammation, particularly as the result of hepatitis B or C virus infection^28^,^29^,^30^. Chronic inflammation following cholecystectomy is considered to associated with an increased risk of developing HCC^2^,^3^,^5^,^31^, but whether it will influence the outcome of HCC patients underwent curative resection remains to be clarified. In this study, stratified analysis of OS according to BCLC staging system demonstrated no significant difference between the cholecystectomy group and no-cholecystectomy group at early, intermediate or advanced stage, and only for patients at early stage, the RFS rates of cholecystectomy group were significantly worse than those of non-cholecystectomy group.

At present, the main cause for the dismal outcome of HCC is the high incidence of recurrence, and the overall prognosis depends largely on the pattern of recurrence^32^. The prognosis of early recurrence is much worse than that of late recurrence because early recurrence is primarily mediated by intrahepatic metastatic mechanism that is associated with tumor vascular invasion and by increased angiogenesis, whereas most of the late recurrences are multicentric occurrence in origin^33^,^34^. In this study, the early recurrence rate of cholecystectomy group was significantly higher than that of no-cholecystectomy group. However, when stratified by BCLC stage, the difference was significant only for patients at early stage, but not for patients at advanced stage.

Further multivariate analysis revealed that cholecystectomy was an independent risk factor for early recurrence but not for late recurrence of early stage HCC patients. It means that the impact of cholecystectomy on postoperative recurrence diminished with increasing time after surgery. It’s interesting that similar phenomenon was discovered in the investigations for risk of developing digestive cancers following cholecystectomy. It’s reported that the rate ratios for developing digestive cancers were significantly high in the first year after cholecystectomy, but decreased rapidly over time and became non-significant as early as two years after cholecystectomy^2^,^35^,^36^,^37^. Since the time-scale of the association is too short, some researchers think that the association between cholecystectomy and subsequent digestive cancer is unlikely to be causal^37^.

However, an additional hypothesis is that cholecystectomy will lead to inflammation and accumulation of bile, both of which can act as carcinogens to promote cancer recurrence. Studies demonstrate that removal of the gallbladder will leads to Sphincter of Oddi (SO) dysfunction, which in turn will cause dilation of the CBD, elevated bile duct pressure, and subsequent cholestasis and chronic inflammation^6^,^7^,^8^,^9^,^10^,^11^. Inflammation-mediated tumor angiogenesis has been confirmed by various studies and release of inflammation related cytokines, chemokines, growth factors plays decisive roles in cancer invasion, recurrence and metastasis^5^,^16^,^17^,^18^. In addition, the accumulation of bile and secondary bile acids, in particular, deoxycholic acid, can act as carcinogens and promote the recurrence and occurrence of cancers^38^,^39^. However, because of internal compensation, SO could adjust to the changes produced by cholecystectomy and SO motility will recover in several months after cholecystectomy to avoid subsequent cholestasis, CBD hypertension and inflammation^40^,^41^. This may not only explains why most patients with postcholecystectomy syndrome recover in a year after operation, but explains the short-term association of cholecystectomy and increased risk of tumorigenesis or recurrence.

There seems to be a consensus among some surgeons that patients with HCC should simultaneously undergo cholecystectomy at the time of resection for HCC, not only because performing a subsequent cholecystectomy on a patient who has undergone prior hepatic surgery is technically difficult^42^, but also for fear of gangrenous cholecystitis after transcatheter arterial chemoembolization (TACE) treatment. Though inadvertent reflux of the embolic material into the cystic artery after TACE has been reported^43^, super selective embolization technique has significantly reduce the risk of cholecystitis and the reported incidence is lower than 5%^44^. In most cases, management can be conservative, and cholecystectomy is not necessary^44^.

In addition, the most common extrahepatic metastatic sites of HCC are lung, abdominal lymph nodes, bone and adrenal gland, direct invasion or metastasis to the gallbladder is rare^42^,^45^. It has been suggested that gallbladder cancer can easily invade the liver because there is no peritoneum between the gallbladder and the liver fossa. On the other hand, HCC does not normally invade the gallbladder because HCC rarely destroys the muscle layer or the collagen fibers of the gallbladder wall^42^. In accordance with the previous study^42^, the prevalence of invasion or metastasis from HCC to the gallbladder was only 0.2% (9/3933) in our study, such low incidence dose does not warrant routine cholecystectomy during resection for HCC unless gallbladder metastasis is suspected.

To the best of our knowledge, no previous study has addressed a possible relation between cholecystectomy and increased risk of early recurrence of early stage HCC. Since cholecystectomy is associated with early recurrence and poorer RFS of early stage HCC patients that underwent curative resection, routine removal of normal gallbladder during HCC resection may be avoided for patients at early stage, and the indication of cholecystectomy may be restricted to patients with gallbladder diseases or metastasis. However, because of the retrospective nature of this study, our findings need to be confirmed by future prospective research before conclusions can be drawn. Though it is too early to consider any potential clinical recommendations, our findings would question the indication of routine cholecystectomy during HCC resection.

### Patients and methods

#### Study population

This study was approved by the Ethic Committee of Zhongshan Hospital and Qilu Hospital, performed in accordance with the approved guidelines, and informed consent was obtained from all subjects.

Data of HCC patients treated by curative resection for HCC in liver cancer institute of Fudan University and Qilu Hospital of Shandong University between January 1999 and December 2009 were retrieved from a prospectively collected database and were retrospectively analyzed. Curative resection was defined as complete resection of all tumor nodules and the cut surface being free of cancer by histologic examination; having no cancerous thrombus in the portal vein, hepatic veins, or bile duct; and having no extrahepatic metastasis^46^,^47^. All pathologic specimens were reviewed by 2 pathologists to confirm the diagnosis of HCC. The histologic grade of tumor differentiation was assigned by the Edmondson grading system. The staging of tumors was determined according to the BCLC staging system. Liver function was assessed by Child-Pugh score system. All the HBV-related HCC patients did not receive any anti-HBV treatment except IFN-a treatment.

Factors that may potentially related to survival and recurrence were selected in this study on the basis of previous studies, including age (≤50 or >50 years), gender (male or female), cirrhosis (yes or no), Child-Pugh Score (A or B), tumor size (≤5 or >5 cm), number of tumor nodules (single or multiple), tumor capsule (yes or no), vascular invasion (yes or no), tumor differentiation (Edmondson’s classification I/II or III/IV), TACE or IFN-α treatment (yes or no). Preoperative laboratory values of serum alanine aminotransferase (ALT) (≤75 or >75U/L), γ-glutamyl transpeptidase (GGT) (≤50 or >50U/L), and a-fetoprotein (AFP) concentration (≤20 or >20 ng/mL) were also included in analysis, using the upper limit of the normal values in our hospital as the cutoff values. All tumor related factors were determined by pathological examination of resected tissue.

#### Follow-up

Patients were followed up every 2 months after operation in outpatient clinics and monitored prospectively for recurrence by a standard protocol that included serum AFP, liver function, and ultrasonography or contrastenhanced computed tomography (CT). CT scan of the abdomen was performed every 6 months. Bone scan or magnetic resonance imaging (MRI) was performed if localized bone pain was reported. Intrahepatic recurrence was defined as a new lesion in the remnant liver with typical imaging appearance in CT/MRI and an elevated AFP level according to EASL proposed criteria for HCC. Ultrasound-guided fine-needle biopsy is sometimes needed to confirm the diagnosis when imaging is atypical. Recurrent cases were divided into early or late recurrent group, using 1 year as the cutoff value, as suggested by Poon’s study^34^. In this study, only the first recurrence was included.

#### Statistical analysis

The χ^2^ test or Fisher’s exact probability was used for categorical variables and Student’s t test was used for continuous variables. Cumulative overall survival rate was calculated by the Kaplan-Meier method and compared by the log-rank test. OS was calculated from the date of resection to the date of death regardless of reason of death. RFS was calculated from the date of resection to the date when tumor recurrence was diagnosed, or from date of the resection to the last visit, if recurrence was not diagnosed, the cases were censored at the date of death or the last date of follow-up. The Cox proportional hazards model was used to determine the independent factors on survival and recurrence. Statistical analyses were performed by SPSS 19.0 (SPSS Inc., Chicago, IL). Two-tailed P < 0.05 was considered statistically significant.

## Additional Information

**How to cite this article**: Li, T. *et al*. Cholecystectomy is associated with higher risk of early recurrence and poorer survival after curative resection for early stage hepatocellular carcinoma. *Sci. Rep.* **6**, 28229; doi: 10.1038/srep28229 (2016).

## Supplementary Material

Supplementary Information

## Figures and Tables

**Table 1:** Tumor staging, operative variables and postoperative outcomes of HCC patients in cholecystectomy and non-cholecystectomy group.

Variable	Non-cholecystectomy (n = 3294)	Cholecystectomy (n = 639)	p value
BCLC stage (%)			<0.001
0/A	2015 (61.2)	307 (48.0)	
B	956 (29.0)	209 (32.7)	
C	323 (9.8)	123 (19.3)	
Resection range (%)			<0.001
Minor (≤2 Couinaud segments)	2288 (69.5)	356(55.7)	
Major (≤2 Couinaud segments)	1006 (30.5)	283(44.3)	
Postoperative complications (%)			
Bile leakage	159(4.8)	35 (5.6)	0.487
Hemorrhage	19 (0.6)	3 (0.5)	0.739
Intra-abdominal abscess	29 (0.9)	8 (1.3)	0.373
Pleural effusion	201 (6.1)	47 (7.4)	0.233
Mean recurrence time(Median), mo	17.9 (12)	13.2 (9)	<0.001
Recurrence, early/late	573/499	165/86	<0.001
BCLC stage 0/A	236/333	59/47	0.007
BCLC stage B	229/141	63/23	0.048
BCLC stage C	108/25	43/16	0.194

**Table 2:** Demographics and clinical characteristics of early stage (BCLC stage 0/A) HCC patients in cholecystectomy and non-cholecystectomy group.

Variables (%)	HCC, n = 2322		
	Non-cholecystectomy, n = 2015	Cholecystectomy, n = 307	p value
Gender			0.220
female	300 (14.9)	54 (17.6)	
male	1715 (85.1)	253 (82.4)	
Age, yrs			0.002
≤50	963 (47.8)	118 (38.4)	
>50	1052 (52.2)	189 (61.6)	
HBsAg			0.811
negative	298 (14.8)	47 (15.3)	
positive	1717 (85.2)	260 (84.7)	
AFP, ng/mL			0.403
≤20	792 (39.3)	113 (36.8)	
>20	1223 (60.7)	194 (63.2)	
AFP, mean, ng/mL	1567.02	1889.07	0.166
≤400	1322 (65.6)	189(61.6)	
>400	693 (34.4)	118 (38.4)	
ALT, U/L			0.115
≤75	1771 (87.9)	260 (84.7)	
>75	244 (12.1)	47 (15.3)	
GGT, U/L			0.117
≤50	910 (45.2)	124 (40.4)	
>50	1105 (54.8)	183 (59.6)	
Cirrhosis			0.608
no	299 (14.8)	49 (16.0)	
yes	1716 (85.2)	258 (84.0)	
Child-Pugh Score			0.157
A	1882 (93.4)	280 (91.2)	
B	133 (6.6)	27 (8.8)	
Tumor size, cm			<0.001
≤5	1317 (65.4)	160 (52.1)	
>5	698 (34.6)	147 (47.9)	
Tumor number			0.260
single	1952 (96.9)	301 (98.0)	
multiple	63 (3.1)	6 (2.0)	
Tumor capsule			0.091
no	1263 (62.7)	177 (57.7)	
yes	752 (37.3)	130 (42.3)	
Tumor differentiation			0.663
I-II	1455 (72.2)	218 (71.0)	
III-IV	560 (17.8)	89 (29.0)	

**Table 3:** Univariate and multivariate analysis of risk factors related to RFS of early stage (BCLC stage 0/A) HCC patients.

Variable	Univariate analysis			Multivariate analysis		
	HR	95% CI	p value	HR	95% CI	p value
Gender						
female	1			1		
male	1.20	0.96–1.50	0.110	1.07	0.85–1.35	0.565
Age years						
≤50	1			1		
>50	1.11	0.95–1.29	0.183	1.15	0.98–1.34	0.091
HBsAg						
negative	1			1		
positive	1.30	1.03–1.62	0.024	1.30	1.02–1.65	0.031
AFP, ng/mL						
≤20	1			1		
>20	1.45	1.24–1.70	<0.001	1.39	1.18–1.64	<0.001
ALT, U/L						
≤75	1			1		
>75	1.23	0.98–1.55	0.068	1.04	0.83–1.31	0.744
GGT, U/L (%)						
≤50	1			1		
>50	1.41	1.21–1.64	<0.001	1.25	1.07–1.47	0.005
Liver cirrhosis						
no	1			1		
yes	1.34	1.07–1.70	0.013	1.24	0.97–1.57	0.083
Child-Pugh Score						
A	1			1		
B	1.29	0.96–1.73	0.094	1.17	0.86–1.57	0.315
Tumor size, cm						
≤5	1			1		
>5	1.42	1.22–1.65	<0.001	1.29	1.10–1.52	0.002
Tumor number						
single	1			1		
multiple	1.02	0.65–1.62	0.922	1.07	0.67–1.70	0.777
Tumor capsule						
yes	1			1		
no	1.22	1.05–1.43	0.011	1.13	0.96–1.32	0.140
Tumor differentiation						
I-II	1			1		
III-IV	1.20	1.02––1.41	0.032	1.08	0.91–1.27	0.343
Vascular invasion						
no	–	–	–	–	–	–
yes	–	–	–	–	–	–
Postoperative TACE (%)						
yes	1			1		
no	1.38	1.34–1.43	<0.001	3.39	2.89–3.99	<0.001
IFN-α treatment						
yes	1			1		
no	1.11	0.86–1.42	0.428	1.22	0.95–1.57	0.121
Cholecystectomy						
no	1			1		
yes	1.25	1.02–1.54	0.034	1.29	1.04–1.59	0.020

HR: Hazard Ratio; CI: Confidence Interval; GGT, γ-glutamyl transferase; ALT, Alanine aminotransferase; AFP, a-fetoprotein.

**Table 4:** Univariate and multivariate analysis of risk factors for early recurrence of early stage (BCLC stage 0/A) patients.

Variable	Univariate analysis			Multivariate analysis		
	HR	95% CI	p value	HR	95% CI	p value
Gender						
female	1			1		
male	1.04	0.75–1.43	0.835	1.05	0.75–1.46	0.779
Age,years						
≤50	1			1		
>50	0.93	0.74–1.17	0.517	1.03	0.81–1.31	0.807
HBsAg						
negative	1			1		
positive	1.23	0.87–1.73	0.243	1.32	0.92–1.91	0.136
AFP, ng/mL						
≤20	1			1		
>20	1.80	1.39–2.32	<0.001	1.54	1.18–2.01	0.001
ALT, U/L						
≤75	1			1		
>75	1.62	1.20–2.19	0.002	1.25	0.92–1.70	0.160
GGT, U/L (%)						
≤50	1			1		
>50	1.80	1.41–2.30	<0.001	1.49	1.16–1.92	0.002
Liver cirrhosis						
no	1			1		
yes	1.16	0.85–1.57	0.356	1.10	0.80–1.52	0.555
Child-Pugh Score						
A	1			1		
B	1.27	0.83–1.94	0.278	1.28	0.83–1.97	0.269
Tumor size, cm						
≤5	1			1		
>5	2.26	1.80–2.85	<0.001	1.78	1.40–2.27	<0.001
Tumor number						
single	1			1		
multiple	1.75	0.72–4.24	0.214	1.37	0.56–3.43	0.493
Tumor capsule						
yes	1			1		
no	1.58	1.26–1.99	<0.001	1.33	1.05–1.69	0.016
Tumor differentiation						
I-II	1			1		
III-IV	1.38	1.09–1.76	0.009	1.19	0.93–1.53	0.157
Vascular invasion						
no	–	–	–	–	–	–
yes	–	–	–	–	–	–
Postoperative TACE (%)						
yes	1			1		
no	3.77	2.94–4.84	3.77	3.67	2.85–4.71	<0.001
IFN-α treatment						
yes	1			1		
no	1.83	1.14–2.95	0.013	1.89	1.17–3.05	0.010
Cholecystectomy						
no	1			1		
yes	1.69	1.27–2.24	<0.001	1.52	1.13–2.03	0.005

HR, Hazard Ratio; CI, Confidence Interval; GGT, γ-glutamyl transferase; ALT, Alanine aminotransferase; AFP, a-fetoprotein.

**Table 5:** Univariate and multivariate analysis of risk factors for late recurrence of early stage (BCLC stage 0/A) patients.

Variable	Univariate analysis			Multivariate analysis		
	HR	95% CI	p value	HR	95% CI	p value
Gender						
female	1			1		
male	1.65	1.08–2.51	0.021	1.41	0.92–2.17	0.119
Age,years						
≤50	1			1		
>50	1.28	0.99–1.66	0.057	1.29	0.98–1.68	0.066
HBsAg						
negative	1			1		
positive	1.16	0.82–1.64	0.399	1.16	0.81–1.67	0.420
AFP, ng/mL						
≤20	1			1		
>20	1.09	0.84–1.41	0.514	1.13	0.86–1.47	0.380
ALT, U/L						
≤75	1			1		
>75	1.23	0.77–1.97	0.388	1.49	0.92–2.39	0.105
GGT, U/L (%)						
≤50	1			1		
>50	1.28	0.99–1.65	0.058	1.17	0.90–1.52	0.255
Liver cirrhosis						
no	1			1		
yes	2.16	1.38–3.38	0.001	2.24	1.40–3.57	0.001
Child-Pugh Score						
A	1			1		
B	1.34	0.78–2.31	0.283	1.40	0.81–2.44	0.230
Tumor size, cm						
≤5	1			1		
>5	1.04	0.79–1.38	0.766	1.07	0.80–1.42	0.659
Tumor number						
single	1			1		
multiple	1.57	0.83–2.96	0.164	1.61	0.84–3.07	0.153
Tumor capsule						
yes	1			1		
no	1.10	0.83–1.45	0.525	1.13	0.85–1.50	0.403
Tumor differentiation						
I-II	1			1		
III-IV	1.15	0.88–1.51	0.317	1.04	0.79–1.38	0.780
Vascular invasion						
no	–	–	–	–	–	–
yes	–	–	–	–	–	–
Postoperative TACE (%)						
yes	1			1		
no	2.91	2.25–3.77	<0.001	2.94	2.20–3.83	<0.001
IFN-α treatment						
yes	1			1		
no	1.37	0.97–1.94	0.074	1.24	0.87–1.77	0.229
Cholecystectomy						
no	1			1		
yes	1.19	0.79–1.77	0.404	1.01	0.67–1.53	0.959

HR, Hazard Ratio; CI, Confidence Interval; GGT, γ-glutamyl transferase; ALT, Alanine aminotransferase; AFP, a-fetoprotein.
