# Supplementary material for: Cholecystectomy is associated with higher risk of early recurrence and poorer survival after curative resection for early stage hepatocellular carcinoma
Source: Sci Rep. 2016 Jun 20;6:28229. doi: 10.1038/srep28229 (PMC4913319; doi:10.1038/srep28229)
Supplement: Supplementary Information [file srep28229-s1.doc]

**Cholecystectomy is associated with higher risk of early recurrence and poorer survival after curative resection for early stage hepatocellular carcinoma**

Tao Li^1*^, M.D, Shu-Kang Wang^2^, M.D, Xu-Ting Zhi^1^, M.D, Jian Zhou^3^, M.D, Zhao-Ru Dong^1^, M.D, Zong-Li Zhang^1^, M.D, Hui-Chuan Sun^3^, M.D, Qing-Hai Ye^3^, M.D, Jia Fan^3^, M.D

Supplementary Table 1. Demographics and clinical characteristics of HCC patients in cholecystectomy and non-cholecystectomy group

| Variables (%) | HCC, n=3933 | |  |
| --- | --- | --- | --- |
|  | Non-cholecystectomy, n=3294 | Cholecystectomy, n=639 | p value |
| Gender |  |  | 0.233 |
| female | 427 (13.0) | 94 (14.7) |  |
| male | 2867 (87.0) | 545 (85.3) |  |
| Age, yrs |  |  | 0.166 |
| ≤50 | 1609 (48.9) | 293 (45.9) |  |
| >50 | 1685 (51.1) | 346 (54.1) |  |
| HBsAg |  |  | 0.186 |
| negative | 455 (13.8) | 101 (15.8) |  |
| positive | 2839 (86.2) | 538 (84.2) |  |
| AFP, ng/mL |  |  | 0.022 |
| ≤20 | 1102 (33.5) | 184 (28.8) |  |
| >20 | 2192 (66.5) | 455 (71.2) |  |
| ALT, U/L |  |  | 0.021 |
| ≤75 | 2870 (87.1) | 535 (83.7) |  |
| >75 | 424 (12.9) | 104 (16.3) |  |
| GGT, U/L |  |  | 0.001 |
| ≤50 | 1280 (38.9) | 204 (31.9) |  |
| >50 | 2014 (61.1) | 435 (68.1) |  |
| Cirrhosis |  |  | 0.029 |
| no | 444 (13.5) | 107 (16.7) |  |
| yes | 2850 (86.5) | 532 (83.3) |  |
| Child-Pugh Score |  |  | 0.574 |
| A | 3067 (91.1) | 591 (92.5) |  |
| B | 227 (8.9) | 48 (7.5) |  |
| Tumor size, cm |  |  | <0.001 |
| ≤5 | 1834 (55.7) | 249 (39.0) |  |
| >5 | 1460 (44.3) | 390 (61.0) |  |
| Tumor number |  |  | 0.570 |
| single | 2762 (83.8) | 530 (82.9) |  |
| multiple | 532 (16.2) | 109 (17.1) |  |
| Tumor capsule |  |  | <0.001 |
| no | 1505 (45.7) | 343 (53.7) |  |
| yes | 1789 (54.3) | 296 (46.3) |  |
| Vascular invasion |  |  | <0.001 |
| no | 2335 (70.9) | 370 (57.9) |  |
| yes | 959 (29.1) | 269 (42.1) |  |
| Tumor differentiation |  |  | 0.132 |
| I-II | 2290 (69.5) | 425 (66.5) |  |
| III-IV | 1004 (30.5) | 214 (33.5) |  |

HR, Hazard Ratio; CI, Confidence Interval;

ALT: alanine aminotransferase; GGT: γ－glutamyltransferase; AFP: a-fetoprotein;

Supplementary Table 2. Multivariate analysis of risk factors for early recurrence of early stage patients underwent minor resection (Only statistically significant factors are listed.)

| Variable | HR | 95% CI | p value |
| --- | --- | --- | --- |
| AFP, ng/mL |  |  |  |
| ≤20 | 1 |  |  |
| >20 | 1.61 | 1.18-2.19 | 0.003 |
| GGT, U/L |  |  |  |
| ≤50 | 1 |  |  |
| >50 | 1.62 | 1.20-2.19 | 0.002 |
| Tumor size, cm |  |  |  |
| ≤5 | 1 |  |  |
| >5 | 1.57 | 1.18-2.10 | 0.001 |
| Postoperative TACE |  |  |  |
| yes | 1 |  |  |
| no | 4.10 | 3.00-5.60 | <0.001 |
| Cholecystectomy |  |  |  |
| no | 1 |  |  |
| yes | 1.47 | 1.00-2.15 | 0.048 |

HR, Hazard Ratio; CI, Confidence Interval; GGT,γ-glutamyl transferase; AFP, a-fetoprotein
